# Simple Purification and Antimicrobial Properties of Bacteriocin-like Inhibitory Substance from *Bacillus* Species for the Biopreservation of Cheese

**DOI:** 10.3390/foods13010010

**Published:** 2023-12-19

**Authors:** Jong-Hui Kim, Eun-Seon Lee, Bu-Min Kim, Jun-Sang Ham, Mi-Hwa Oh

**Affiliations:** Animal Products Utilization Division, National Institute of Animal Science, RDA, Wanju 55365, Republic of Korea; kimjh8199@korea.kr (J.-H.K.); les1023@korea.kr (E.-S.L.); scarlet7@korea.kr (B.-M.K.); hamjs@korea.kr (J.-S.H.)

**Keywords:** *Bacillus*, bacteriocin-like inhibitory substance, aqueous two-phase system

## Abstract

Bacteriocins may be used as natural preservatives and antibiotic substitutes in various foods. However, the multistep purification process of bacteriocins results in high production costs, which is an obstacle to their commercial use and consumer accessibility. In this study, a bacteriocin-like inhibitory substance (BLIS) from *Bacillus* spp. isolated from Korean fermented foods was partially purified using the aqueous two-phase system (ATPS). The maximum activity of the BLIS was achieved for ATPS composed of PEG 1000 (15% [*w*/*w*])/ammonium sulfate (20% [*w*/*w*])/sodium chloride (2% [*w*/*w*]), which caused BLIS activity to increase by 3 times with a 99% recovery rate. In particular, *B. amyloliquefaciens* Y138-6 BLIS exhibited broad antibacterial activity, high resistance to acid-base stress, and excellent thermal stability. This antibacterial substance inhibited the growth of aerobic bacteria and fungi on the walls of cheese and ripening rooms. These antibacterial properties have been shown to increase food safety and have the potential for use as biopreservatives. Moreover, considering that the execution of the ATPS requires only salts and PEG, it is a simple, environmentally friendly, and cost-effective process and may have industrial applications in the recovery of BLIS from fermentation broth.

## 1. Introduction

The rising demand for food safety among consumers has spurred a growing interest in natural preservatives. In dairy processing applications, the use of bacteriocins has become an attractive prospect owing to their safety and environmentally friendly properties [1].

Microorganisms, including bacteria, yeast, and fungi, are present in cheese throughout the ripening process. Some of them contribute to ripening in a positive way [2]. However, they can also cause spoilage or produce other metabolites that reduce the undesirable aromas, flavors, or quality of the cheese. Moreover, spoilage and the outgrowth of pathogenic bacteria, such as *Escherichia coli*, *Listeria monocytogenes*, *Salmonella enterica*, and *Staphylococcus aureus*, can cause gastrointestinal and zoonotic diseases [3,4]. Notably, some fungi known to cause food spoilage can produce mycotoxins that are potentially dangerous to consumers [5]. Among these, *Penicillium* spp. are typically the main culprits behind the spoilage of ripened cheese [6,7]. Chemical preservatives, such as sorbates, propionate, and natamycin, are used to control spoilage microorganisms [5]. However, they negatively impact human health and the nutritional value of food [8]. Therefore, there is growing interest in using bacteriocins as biological agents that could replace chemical preservatives.

Bacteriocins are ribosomally synthesized peptides that exert antibacterial effects against strains of the same species or species more distantly related to the bacteriocin-producer [9]. Bacteriocin-like inhibitory substance (BLIS) is a term used for not yet fully characterized antimicrobial peptides or proteins that exert bactericidal or bacteriostatic effects against Gram-positive and Gram-negative bacteria without affecting the producer [9]. These typically have different chemical structures and contain unusual amino acids [9]. As these compounds are of natural origin and are stable based on heat and pH, they are considered a potential means of preserving food safety [7,8,9]. Commonly employed purification methods include organic solvent precipitation, centrifugation, dialysis, chromatography, and filtration. Multistep purification processes have inherent disadvantages, including high production costs, low yields, and skilled operator requirements [10]. The required bacteriocin purity primarily depends on the application and safety of the purified product [11,12]. Therefore, highly purified bacteriocins may not be required for food preservation [13].

The aqueous two-phase system (ATPS) is attracting attention in the field of the purification of various biomolecules (e.g., proteins, enzymes, antibodies, and DNA) because it provides a mild environment for the extraction of biological materials that are sensitive to external stimuli [14]. In addition, because this method can directly extract BLIS from the fermentation medium, the purification process can be simplified. In this study, the optimal partitioning conditions for ATPS were used to partially purify BLIS from *Bacillus* spp. and to verify their potential to control microbial contaminants during Gouda cheese ripening.

## 2. Materials and Methods

### 2.1. Materials

Polyethylene glycol (PEG) with molar masses of 1000, 3000, 4000, and 10,000 g/mol were purchased from Sigma-Aldrich (Seoul, Republic of Korea). The salts, sodium citrate (Na_3_C_6_H_5_O_7_), ammonium sulfate [(NH_4_)_2_SO_4_], potassium phosphate (KH_2_PO_4_), and sodium chloride (NaCl), were also purchased from Sigma-Aldrich. Tryptic soy, potato dextrose, and mannitol-yolk-polymyxin B broths were purchased from BD Difco (Franklin Lakes, NJ, USA). The bicinchoninic acid (BCA) protein assay kit was supplied by Thermo Fisher Scientific (Pierce, #23227; Waltham, MA, USA). All chemicals used in this study were of analytical grade.

### 2.2. Isolation and Identification of Bacillus Species

In total, 904 *Bacillus* isolates, except *B. cereus*, were isolated from 79 soy sauce (374 isolates), 111 kimchi (336 isolates), 96 pickle (173 isolates), and 9 salted fish samples (21 isolates). To identify the isolates, the 16S rDNA of these strains was amplified with the universal bacterial primers 27F (5′-AGAGTTTGATCCTGGCTCAG-3′) and 1492R (5′-GGTTACCTTGTTACGACTT-3′), and the following PCR conditions were used: 94 °C for 5 min; 30 cycles at 94 °C for 1 min, 55 °C for 1 min, and 72 °C for 1.5 min; final extension at 72 °C for 5 min [15]. The resulting PCR products were purified using a Solg Gel & PCR Purification Kit (Solgent, Daejon, Republic of Korea) and thereafter sequenced by Solgent. The derived sequences were compared with known bacterial sequences in the NCBI GenBank database using BLASTn.

### 2.3. Screening of Isolates for Antimicrobial Activity

Antimicrobial activity of the *Bacillus* isolates was evaluated using the agar well diffusion method, with some modifications [16]. The cell-free supernatants were separated via centrifugation at 12,000× *g* using a refrigerated centrifuge at 4 °C and filtered using 0.22 µm pore filter membranes (Millipore, Molsheim, France) to remove residual bacterial cells.

The supernatants (100 μL) were placed in wells (6 mm diameter) of 0.75% (*w*/*v*) TSA soft agar plates. The plates were incubated at 37 °C for 24 h for the growth of five foodborne pathogenic bacteria (*B. cereus* KCCM 40935, *L. monocytogenes* CCARM 0214, *E. coli* O157:H7 ATCC43888, *Salmonella enteritidis* KCCM12021, and *S. aureus* ATCC 11778) and *Penicillium* sp. (isolated from this Lab: Animal Products Utilization Division, National Institute of Animal Science, RDA, Wanju, Republic ok Korea) as the target microorganisms. Subsequently, the diameters of the growth inhibition zones were measured using a digital caliper gauge (model 684132; Lee Tool, Ludlow, MA, USA), and the antimicrobial activity was defined in arbitrary units (AU), which is the unit area of the inhibition zone per unit volume. This was calculated using Equation (1).

(1) $$\mathrm{Antimicrobial}\;\mathrm{activity}\left(\frac{\pi R^{2}}{mL}\right)=\frac{Lz-Ls}{V}=\frac{AU}{mL}$$

where Lz = clear zone area (mm^2^), Ls = well area (mm^2^), and V = volume of sample (mL). All experiments were performed in triplicate, with the average values presented.

### 2.4. Determination of Optimum Conditions for Growth and BLIS Production

Selected *Bacillus* isolates were used to inoculate 100 mL aliquots of tryptic soy broth, followed by incubation at 30 °C for 12–18 h. Various temperatures (25, 30, 37, and 45 °C) and initial pH values (4, 5, 7, and 8) were evaluated to explore the optimal growth and BLIS production. To monitor bacterial growth, an aliquot of the culture was removed at time intervals of 0, 6, 12, 18, 24, 30, 36, 24, 48, and 72 h at 30 °C, and the absorbance was measured at 600 nm. Simultaneously, BLIS activity against *S. aureus* was confirmed using the agar well diffusion method, as previously described in Section 2.3.

### 2.5. Determination of Total Protein Concentration

The total protein concentration was determined using a BCA protein quantification kit (Beyotime, Jiangsu Kaiji Biotechnology, Haimen, China), which is compatible with the polymers used in this study [17]. Samples containing proteins (100 μL) and 2 mL of BCA working reagent, prepared according to the manufacturer’s instructions, were added to a test tube. After 30 min, the optical density was determined at 562 nm using a spectrophotometer, with deionized water as the blank. Absorbance values were correlated with the protein concentration based on a calibration curve using bovine serum albumin solutions from 0 to 1000 μg/mL (equation obtained: y (ABS) = 0.0011 × (μg/mL) + 0.0245, R^2^ = 0.99).

### 2.6. Optimal Conditions for Partial Purification of BLIS via the Aqueous Two-Phase System

#### 2.6.1. Effect of Different Constructions of the Aqueous Two-Phase System

The ATPS preparation was prepared according to Lappe et al. (2012) [12], with some modifications. To avoid diluting the samples, extraction experiments in the ATPS were performed at 25 °C by directly dissolving PEG and the salt in the culture supernatant. The *Bacillus* culture supernatant (2.5 mL) was mixed with the ATPS using a vortex for 30 s to form a system with a final concentration of 20% (*w*/*v*) PEG and salts and a total system volume of 5 mL. Phase separation was achieved through centrifugation at 2860× *g* for 10 min. Finally, the volumes of both phases were measured, and samples from the coexisting phases were obtained for antimicrobial and protein assays.

Specific activity, defined as the ratio between antimicrobial activity (AU) in the phase sample and the total protein concentration (mg), was calculated using Equation (2).

(2) $$\mathrm{Specific}\;\mathrm{activity}=\frac{\mathrm{Antimicrobial}\;\mathrm{activity}\left(AU\right)}{\mathrm{Protein}\;\mathrm{concentration}\left(mg\right)}$$

The purification factor was calculated as the ratio of BLIS-specific activity in the top phase to that in the original supernatant, as shown in Equation (3):

(3) $$\mathrm{Purification}\;\mathrm{factor}=\frac{\mathrm{Specific}\;\mathrm{activity}\;\mathrm{of}\;\mathrm{top}\;\mathrm{phase}}{\mathrm{Specific}\;\mathrm{activity}\;\mathrm{of}\;\mathrm{the}\;\mathrm{original}\;\mathrm{supernatant}}$$

The protein yield (%) was calculated using Equation (4):

(4) $$\mathrm{Yield}\left(\%\right)=\frac{\mathrm{protein}\;\mathrm{of}\;\mathrm{top}\;\mathrm{phase}}{\mathrm{protein}\;\mathrm{of}\;\mathrm{supernatant}}$$

#### 2.6.2. Effect of PEG Molecular Weight and Concentration

The PEG molecular weight and concentration used in this study were based on the method of Park and Khang (2010) [18], with some modifications. To study the effect of PEG on the partitioning of BLIS from *Bacillus* spp. using the ATPS, different molecular weights (1000, 3000, 4000, and 10,000) of PEG at different concentrations (10, 15, 20, and 25% [*w*/*w*]) were mixed with 20% (NH_4_)_2_SO_4_ in an aqueous system.

#### 2.6.3. Effect of Salt Type and Concentration

The salt type and concentration were prepared using the method of Lappe et al. (2012) [12], with some modifications. To study the effect of salts on the partitioning of BLIS from *Bacillus* spp. using the ATPS, different salts, including Na_3_C_6_H_5_O_7_, (NH_4_)_2_SO_4_, and KH_2_PO_4_, at different concentrations (15, 20, and 25% [*w*/*w*]) were mixed with 15% PEG 1000 in an aqueous system.

#### 2.6.4. Effect of NaCl Concentration

The NaCl concentration was prepared according to Park and Khang (2010) [18], with some modifications. To study the effect of NaCl on the partitioning of BLIS from *Bacillus* spp. using the ATPS, different concentrations (0, 2, 4, 6, and 8% [*w*/*w*]) of NaCl were mixed with 15% PEG 1000/20% (NH_4_)_2_SO_4_ in an aqueous system.

### 2.7. Thermal and pH Stability

Thermal and pH stability were measured as previously described [19], with slight modifications. The tolerance of the BLIS, partially extracted using the ATPS, at different temperatures and pH values was measured. To determine thermal stability, BLIS of three isolates was exposed to different temperatures (50, 60, 70, 80, 90, 100, and 121 °C [autoclaving]) for 15 min. Untreated BLIS was used as a control. The relative antimicrobial activity was assessed using the agar well diffusion method (Section 2.3), and the zone of inhibition was measured in millimeters.

To evaluate the pH stability of the BLIS, buffers were prepared by adjusting the pH of BLIS with dilute HCl and NaOH (Sigma-Aldrich, St. Louis, MO, USA). Samples at different pH values (from pH 3–9) were incubated at 37 °C for 2 h. Antimicrobial activity against the indicator strain, *S. aureus*, was determined using the BLIS activity described above and expressed as relative antibacterial activity.

### 2.8. Determination of Minimum Inhibitory and Bactericidal Concentrations

The inhibitory effect of BLIS against *S. aureus* was tested in accordance with the results of Xiang et al. [20], with slight modifications. BLIS was diluted to various concentrations (1.25, 2.5, 5, 7.5, 10, and 30 mg/mL) and used to determine the minimum inhibitory concentration (MIC) and minimum bactericidal concentration (MBC) via the broth microdilution assay. The plates were incubated at 37 °C for 24 h, whereafter the MIC was calculated via spectrophotometry (absorbance at 620 nm) and visual observations. The lowest concentration at which no bacterial colonies were observed was considered the MBC.

### 2.9. Cytotoxicity Assessment

The 3-(4,5-dimetildiazol-2-yl)-2,5 diphenyltetrazolium (MTT) assay was used to evaluate the toxicity of *Bacillus* isolate BLIS [21]. Caco-2 cells were seeded at a density of 10^5^ cells/well in 24-well plates (SPL Life Sciences, Pocheon, Republic of Korea) and incubated for 16–20 h (37 °C, 5% [*v*/*v*] CO_2_) until monolayers formed. The cells were treated with each of the three BLISs at concentrations of 50–150 mg/mL and incubated for 24 h. After this period, the cells were analyzed using a water-soluble MTT assay using the EZ-CYTOX kit (Daeillab, Seoul, Republic of Korea), according to the manufacturer’s instructions. Cell survival was assessed by measuring the optical density at 450 nm.

### 2.10. Application of BLIS on Cheese Surfaces and Ripening Room Walls

Gouda cheese for BLIS application was prepared according to the method described by Park et al. [22]. Ten milliliters of BLIS (5 mg/mL) was evenly sprayed onto the surface of 10 cm^2^ Gouda cheese (ripened for 3 months) and 10 cm^2^ of the walls of a ripening room. The BLIS activity was assessed based on the number of microorganisms remaining over time. At selected times (0, 1, 2, 4, and 7 days) following BLIS treatment, the surface of the cheese was swabbed using a sterile cotton tip, which was then smeared onto an agar plate, and the number of remaining aerobic bacteria and fungi was counted. Untreated Gouda cheese was used as the control. In addition, the reduction level of *Staphlococcus* and *Bacillus* was confirmed by counting colonies grown on Baird–Parker agar (BPA) and Mannitol Egg Yolk (MYP) Agar.

### 2.11. Statistical Analysis

All data are presented as the mean ± standard deviation of triplicate samples. Statistical significance between treatments was determined using Student’s *t*-tests with IBM SPSS Statistics version 26 (SPSS Inc., Chicago, IL, USA). The differences were considered statistically significant when *p* < 0.05.

## 3. Results

### 3.1. Isolation and Identification of BLIS-Producing Bacillus Species

**Figure 1:**
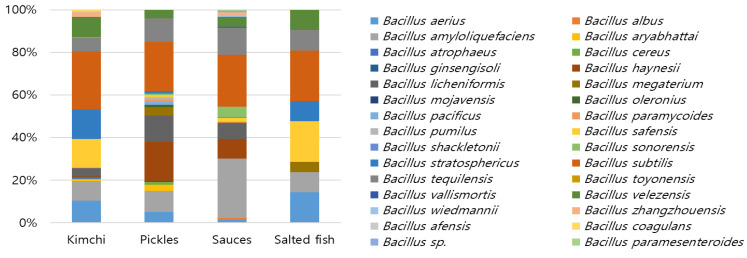
Distribution of *Bacillus* species diversity as determined in different Korean fermented foods. Kimchi, *n* = 111; pickles, *n* = 96; sauces, *n* = 79; and salted fish, *n* = 9.

The microbiota present in 295 different samples of Korean fermented foods were explored, focusing on *Bacillus* spp., with potential biopreservation properties. Thirty *Bacillus* spp. were detected in all fermented foods, with *B. subtilis* and *B. amyloliqufaciens* being the dominant species detected, comprising 42.2% of the total population (Figure 1). The antibacterial activity of 904 isolated *Bacillus* strains was evaluated using the agar well diffusion method, and the ability of *Bacillus* to produce BLIS and its effectiveness as an antibacterial agent were evaluated. As shown in Table 1, all the indicator strains, except for *Salmonella enteritidis*, were sensitive to BLIS, exhibiting large diameters of the inhibition halos (18.31 to 22.89 mm), regardless of the BLIS dilution. The Y120-8, Y138-6, and Y167-2 strains were ultimately chosen because of their significant anti-*Listeria* activity, as well as their inhibitory effects against *B. cereus*, *S. aureus*, and *Penicillum* sp., the major spoilage microorganisms of cheese. In particular, unlike Y120-8 and Y167-2, which only inhibited Gram-positive bacteria, the Y138-6 isolate showed high antibacterial activity against Gram-negative *E. coli.* Three isolates were identified through 16S rDNA sequencing, with Y120-8 being identified as *B. velezensis* (99.8% 16 s rRNA gene sequence similarity), Y138-6 as *B. amyloloquefaciens* (99.9%), and Y167-2 as *B. subtilis* (99.9%).

### 3.2. Bacillus Species Growth, BLIS Production, and Influence of Growth Temperature

**Figure 2:**
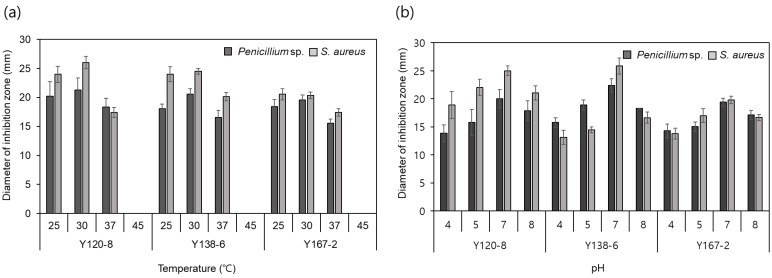
(**a**) Optimal temperature and (**b**) pH for BLIS production in *Bacillus* isolates in tryptic soy broth medium.

To determine the optimal conditions for BLIS production based on the growth kinetics results (Table 2), the timing of BLIS release according to the growth of the isolates and BLIS activity according to temperature and initial pH (Figure 2) were explored. BLIS production at 30 °C was higher than that at 25 and 37 °C. No bacterial growth was observed at 45 °C. The optimal pH of BLIS production was 7 for all three strains. Growth kinetic results showed that increases in BLIS activity were dependent on an increase in the growth rate. BLIS production by the three tested strains began in log phase. The maximum growth levels (optical density values of approximately 0.8) and inhibition zone diameters (21–23 mm) were achieved by culturing for 42–48 h in tryptic soy broth at 30 °C. Therefore, the optimal culture conditions for BLIS production are 30 °C, pH 7, and 42–48 h, and subsequent experiments were conducted under these conditions.

### 3.3. Optimal Aqueous Two-Phase System Conditions for Purification of BLIS

BLIS partitioning in different types and concentrations of PEG/salts with different amounts of NaCl are shown in Table 3. Selecting a polymer with an ideal molecular weight is a crucial step in the construction of an ATPS. The influence of different molecular weights of PEG in combination with (NH4)_2_SO_4_ was initially tested to evaluate the recovery of BLIS. The highest BLIS activity was recorded for PEG 1000 at 15% (*w*/*w*) (410.6 ± 17.4~817.2 ± 53.6 AU/mg) in all three *Bacillus* spp. Under the influence of various inorganic salts, the maximum BLIS activity (363.3 ± 42.21 to 810.5 ± 28.2 AU/mL) was obtained for systems containing 20% (*w*/*w*) (NH_4_)_2_SO_4_. Considering that the PEG/(NH4)_2_SO_4_ system resulted in high specific activity and purification, the effect of the addition of NaCl to the system was investigated.

The addition of 2% NaCl increased the specific activity and purification factor (Table 3). However, at high concentrations (4% and 6%) of NaCl, the specific activity and purification factor decreased, while the protein yield increased. Overall, the optimal ATPS condition for BLIS extraction from *Bacillus* cultures was PEG 1000 (15% [*w*/*w*])/(NH_4_)_2_SO_4_ (20% [*w*/*w*])/NaCl (2% [*w*/*w*]), which resulted in BLIS activity increasing by three times with a yield of more than 99%.

### 3.4. Effect of Temperature and pH on BLIS Activity

The effects of temperature and pH on the antibacterial activity of the BLIS of the three *Bacillus* isolates are presented in Table 3. A stability study of partially purified BLIS at various temperatures revealed that the BLIS of *B. amyloliquefaciens* Y138-6 was stable up to 90 °C; however, its activity decreased at temperatures above 100 °C. The inhibitory activity of *B. velezensis* Y120-8 and *B. subtilis* Y167-2 decreased by 19% and 13%, respectively, at 70 °C, and all three BLIS types were completely inactivated after steam sterilization. BLIS from all tested isolates maintained antibacterial activity in the range of 92–100% at pH 4–8. However, the activity decreased by averages of 11% at pH 3 and 9 and 23% at pH 2 (Table 4).

### 3.5. In Vitro Cytotoxicity Evaluation of BLIS

**Figure 3:**
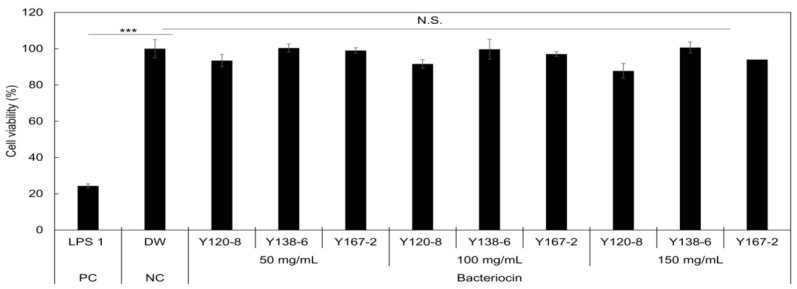
Cytotoxicity of partially purified BLIS from *Bacillus* isolates on Caco-2 cells in vitro. Data were expressed as the mean ± standard deviation (*n* = 5). N.S. = no significance when compared with the negative control group (DW); Student’s *t*-test. *** *p* < 0.001.

The cytotoxic effects of BLIS from the three *Bacillus* isolates on Caco-2 cells were evaluated using an MTT assay, as shown in Figure 3. The results showed no significant differences between the cell viability values (measured at 570 nm) after treatment with different BLIS concentrations when compared with that of the negative control (DW). Cell viability exceeded 87%, suggesting that the partially purified BLIS at the tested concentrations was not cytotoxic to Caco-2 cells (*p* < 0.05). Although no marked cytotoxicity was observed, the percentage of viable cells exposed to the three *Bacillus* BLISs decreased to 93.4%, 91.5%, and 87.7%, respectively, as the concentration of Y120-8 increased. In the case of Y138-6, no cytotoxicity was observed, even at a high concentration (150 mg/mL).

### 3.6. Application of BLIS to Cheese Surfaces and Ripening Room Walls

The *B. amyloliqufacience* Y138-6 strain showed the broadest antibacterial activity among the tested isolates and the highest BLIS-specific activity (832.5 AU/mg). Therefore, the antimicrobial effects of the Y138-6 BLIS were evaluated in ripened Gouda cheese and a ripening room.

**Figure 4:**
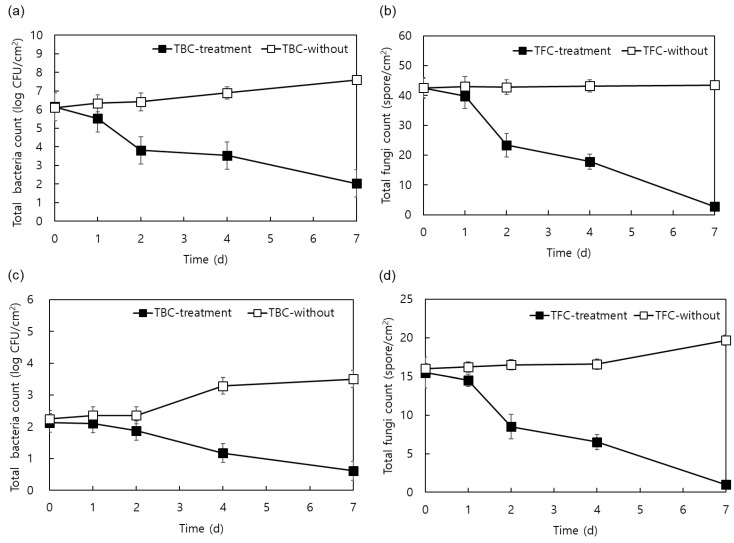
Changes in the number of (**a**) aerobic bacteria and (**b**) fungi on the surface of Gouda cheese and the number of (**c**) aerobic bacteria and (**d**) fungi in the cheese-ripening room walls after exposure to partially purified Y138-6 BLIS.

First, the total numbers of aerobic bacteria and fungi on the surface of cheese (ripened for 3 months) stored in a ripening room, as well as the ripening room walls, were investigated. Averages of 6-log CFU/cm^2^ aerobic bacteria and 42.5 spore/cm^2^ fungi were present on the surface of the Gouda cheese, and averages of 2-log CFU/cm^2^ aerobic bacteria and 15.5 spore/cm^2^ fungi were present on walls of the ripening room. After treating the surface of the same cheese sample with Y138-6 BLIS, the number of microorganisms gradually decreased over time, and the number of viable aerobic bacteria and fungi on day 7 of storage was approximately 2-log CFU/cm^2^ and 2.7 spore/cm^2^, respectively (Figure 4a,b). In the cheese-ripening room, where contamination was low compared with that on the cheese surface, the numbers of aerobic bacteria and mold were less than 5 CFU/cm^2^ or spore/cm^2^ on day 7 of storage (Figure 4c,d). However, aerobic bacteria and fungi in the cheese and ripening room, that were not treated with BLIS, increased by an average of 1.4 log CFU/cm^2^ and 3.5 spore/cm^2^, respectively, from the initial contamination level.

## 4. Discussion

*Bacillus* spp. grow easily on inexpensive nutritional sources, and some have been designated as Generally Recognized As Safe (GRAS) by the Food and Drug Administration (FDA) [23]. Therefore, these species and their products, which have been confirmed as safe, can be used in the food industry. *Bacillus* spp. are producers of bacteriocins or BLIS, and numerous species possess antibacterial activity against pathogens [24,25,26]. In the present study, BLIS from *Bacillus* isolates in Korean fermented foods was partially purified using the ATPS method, and their applicability in cheese preservation was confirmed. The ATPS used in this study is of particular interest because the polymers it comprises are nontoxic, biodegradable, and approved by the FDA. Furthermore, they have previously been used to solubilize and stabilize pharmaceutical and biomedical products [14].

In this study, the BLIS isolated from three *Bacillus* isolates showed strong inhibition zones against the growth of foodborne pathogens, including *S. aureus*, *L. monocytogenes, B. cereus*, and *Penicillium* sp. Moreover, Y138-6 inhibited the growth of *E. coli*, a Gram-negative organism, which has thus far only been reported for a few bacteriocins produced by *Bacillus* spp. [20,27]. Although bacteriocins primarily exhibit inhibitory activity against Gram-positive pathogens, the antagonistic effect of bacteriocins on Gram-negative bacterial spoilage may be related to the production of organic acids [28]. However, in this study, the supernatants of the three strains, including Y138-6, had a pH close to 7, and the activity was completely lost when treated with the proteolytic enzymes proteinase K, protease, and trypsin (2 mg/mL). These findings suggest that the *Bacillus* isolates produce BLIS and that proteins or peptides are responsible for the antibacterial activity. Therefore, the ability of *B. amyloliquefacience* Y138-6 to inhibit the growth of diverse foodborne pathogens suggests that the BLIS produced by this strain may be useful as biological control agents in food production.

Antibacterial activity assessments related to partial purification of the optimal culture and BLIS for the production of antibacterial substances from *Bacillus* isolates were performed using *S. aureus* as an indicator organism owing to its high contamination rates in cheese (Figure 2). Growth kinetic studies revealed that the three isolated *Bacillus* cultures entered the late exponential phase within 30 h at 30 °C and then stationary phase for the 72 h tested. Antibacterial substances were rapidly produced from 12 to 18 h and maintained for up to 72 h.

To economically produce bacteriocin from *Bacillus* spp., research is needed to isolate bacteriocin from the fermentation broth. Typically, biological processes for bacteriocin extraction are complex, accounting for approximately 60–90% of the cost spent on downstream processing. However, the low interfacial tension, selective separation, and the continuous mode of the ATPS enable high yields through solvent extraction. The PEG/salt system has proven to be a convenient method for the rapid extraction of nisin [29]. The present study employed an ATPS with a polymer (PEG)/salt ([NH_4_]_2_SO_4_) to isolate BLIS from the fermentation broth of *B. velezensis* Y120-8, *B. amyloliquefaciens* Y138-6, and *B. subtilis* Y167-2. When an ATPS is prepared by adding PEG and salt to the fermentation broth, BLIS with a hydrophobic surface moves to the top layer (PEG-rich phase), and microbial cells remain in the bottom layer (salt-rich phase). The PEG/salt ATPS is significantly affected by the molecular weight, concentration of PEG, and salt type and depends on the type of target substance to be extracted [18,30].

In this study, the optimal molecular weight of PEG for BLIS extraction was investigated. As the molecular weight of PEG increased, layer separation of the aqueous phase occurred more rapidly due to increasing hydrophobicity. However, the best BLIS extraction was achieved with 15% PEG 1000, which has a low molecular weight, for all three *Bacillus* spp. Similar results were reported in a study on BLIS extraction from *Streptococcus parauberis* Z49 by Back and Khang [18]. However, the PEG/salt systems used in the current study mainly consist of PEG 2000 [31], PEG 4000 [29], and PEG 8000 [32], which have high molecular weights. This difference can be explained by variations in the number of amino acid residues exposed on the surfaces of different types of bacteriocins [33].

In a study on the effects of different salts and their concentration in combination with PEG, the ATPS containing 20% (NH_4_)_2_SO_4_ showed maximum antibacterial activity and yield values (Table 3). No phase separation was observed when 15% (NH_4_)_2_SO_4_ was used. Cascone et al. [34]. reported that adding NaCl, an electrolyte material, to a PEG/salt ATPS can increase the hydrophobicity of the distribution coefficient of the target protein toward the PEG phase. Lappe et al. [12] increased the yield and purity of cerein 8A from *B. cereus* by adding 1 M NaCl. Similarly, in our study, the addition of 2% NaCl led to maximum BLIS activity observed. In contrast, BLIS activity rapidly decreased with the addition of 6% NaCl, although the yield increased. This is believed to be because high concentrations of NaCl cause the denaturation of bacteriocins [33]. Based on these results, the highest BLIS yield and purity were achieved with a system containing 15% PEG 1000, 20% (NH_4_)_2_SO_4_, and 2% NaCl. Under these conditions, purification was approximately three times higher with a yield of more than 99%.

The antibacterial activity of BLIS from *B. amyloliquefaciens* Y138-6 against *S. aureus* was stable and resistant to heat treatment at up to 90 °C. Previous studies also support that bacteriocins from *B. amyloliquefaciens* are thermostable [16,21]. In addition, the Y138-6 BLIS maintained high antibacterial activity from pH 3 to 8 and showed wide pH resistance. This evidence indicates that the Y138-6 BLIS is highly resistant to acid–base stress and has excellent thermal stability, making it a promising biopreservative candidate for heat, acidic, and basic food processing. In addition, this BLIS was shown to be safe in a cytotoxicity test using Caco-2 cells (Figure 3).

The surface of the cheese used in this study was naturally contaminated with microorganisms during ripening, and genetic analysis showed that *Staphylococcus* and *Bacillus* accounted for more than 50% of the bacteria present, followed by *Escherichia*. In the mycoflora analysis, *Penicillium* was the most abundant fungus, followed by *Aspergillus*.

The MIC and MBC of the partially purified Y138-6 BLIS recorded for *S. aureus* growth inhibition were 1.25 and 5 mg/mL, respectively. To apply this antibacterial substance to cheese and facilities, the final concentration of partially purified BLIS was set to 5 mg/mL and was sprayed onto the surface of Gouda cheese that was aged for 3 months and the walls of the ripening room in which the cheese was stored. The application of partially purified Y138-6 BLIS to the cheese surface resulted in a 4-log reduction in viable aerobic bacteria after 7 days at 13 °C. Additionally, viable aerobic bacteria or fungi on the walls of the ripening room, which were not initially heavily contaminated, were hardly detected after 7 days. To confirm the presence of *Staphlococcus* and *Bacillus* among the bacteria surviving after Y138-6 BLIS treatment, Baird–Parker agar (BPA, *Staphlococcus* selection medium) and Mannitol Egg Yolk (MYP) Agar (*Bacillus* selection medium) were used. Consequently, less than 1 log CFU/mL was detected for both strains on both the cheese surface and the ripening room. This demonstrates that the partially purified BLIS of *B. amyloliquefaciens* Y138-6, which has a broad antibacterial spectrum and inhibits the growth of both bacteria and mold, could be used as a protective agent to increase the safety of cheese.

*Staphylococcus* is an opportunistic pathogen frequently detected in dairy farms [35]. Milk is a good substrate for the growth of *S. aureus* and *B. cereus*, and dairy products are known sources of toxicity [36,37]. *Penicillium*, *Cladosporium*, and *Aspergillus* were the most frequently occurring fungi in cheese-ripening rooms [38]. In this regard, further research is necessary to determine the effect of BLIS on the major cheese spoilage microorganisms *S. aureus*, *Cladosporium*, and *Aspergillus*.

## 5. Conclusions

Using the ATPS approach, BLIS from *B. velezensis* Y120-8, *B. amyloliquefaciens* Y138-6, and *B. subtilis* Y167-2 strains, isolated from Korean fermented foods, was partially purified. The proposed approach has high industrial potential for the recovery of BLIS from fermentation cultures through a single process. The BLIS activity partially purified using the ATPS increased 3.1 times, with a recovery rate higher than 99%. Moreover, *B. amyloliquefaciens* Y138-6 BLIS inhibited the growth of aerobic bacteria and fungi on cheese surfaces and ripening room walls. These antibacterial properties have been shown to enhance food safety and may have applications as biopreservatives. In future studies, tricine-SDS-PAGE should be performed to check the purity and estimate the molecular weight of partially purified BLIS.

## Figures and Tables

**Table 1:** Inhibitory spectrum of *Bacillus* spp. isolated from fermented foods.

Indicator Strains	Isolates		
	*Bacillus velezensis* Y120-8	*Bacillus amyloliquefaciens* Y138-6	*Bacillus subtilis* Y167-2
*Staphylococcus aureus*	19.97 ± 0.5	24.03 ± 0.4	22.24 ± 0.5
*Listeria monocytogenes*	18.31 ± 0.2	18.88 ± 0.7	18.65 ± 0.3
*Bacillus cereus*	20.58 ± 0.3	22.89 ± 0.5	20.03 ± 0.1
*Penicillium* sp.	19.58 ± 0.6	21.13 ± 0.5	21.32 ± 0.5
*Salmonella enteritidis*	-	-	-
*Escherichia coli*	-	21.78 ± 0.37	-

The values are the mean values of the diameters of the inhibition halos and were measured in millimeters.

**Table 2:** Growth dynamics and antimicrobial activity of Y120-8, Y138-6, and Y167-2 isolates against *Staphylococcus aureus*.

Time (h)	*Bacillus velezensis* Y120-8		*Bacillus amyloliquefaciens* Y138-6		*Bacillus subtilis* Y167-2	
	OD_600_	Diameter of Inhibition Zone (mm)	OD_600_	Diameter of Inhibition Zone (mm)	OD_600_	Diameter of Inhibition Zone (mm)
6	0.1 ± 0.0	0.0 ± 0.0	0.1 ± 0.0	0.0 ± 0.0	0.1 ± 0.0	0.0 ± 0.0
12	0.2 ± 0.0	16.1 ± 0.8	0.4 ± 0.0	14.4 ± 0.1	0.2 ± 0.0	0.0 ± 0.0
18	0.2 ± 0.0	17.4 ± 0.3	0.4 ± 0.0	17.9 ± 0.1	0.3 ± 0.0	17.9 ± 0.2
24	0.4 ± 0.1	20.4 ± 0.6	0.5 ± 0.1	20.1 ± 0.8	0.4 ± 0.0	19.0 ± 0.5
30	0.7 ± 0.1	20.6 ± 0.7	0.6 ± 0.0	20.7 ± 0.1	0.7 ± 0.1	21.2 ± 0.1
36	0.8 ± 0.1	21.6 ± 0.3	0.6 ± 0.1	20.7 ± 0.4	0.7 ± 0.0	21.7 ± 0.4
42	0.8 ± 0.1	21.5 ± 0.1	0.5 ± 0.1	21.1 ± 0.1	0.8 ± 0.0	21.8 ± 0.3
48	0.9 ± 0.0	22.0 ± 0.1	0.7 ± 0.0	21.3 ± 0.4	0.8 ± 0.0	23.0 ± 0.0
72	0.8 ± 0.1	22.3 ± 0.2	0.9 ± 0.0	21.4 ± 0.1	0.8 ± 0.0	22.7 ± 0.4

**Table 3:** Effect of different constructions of the aqueous two-phase system on partially purified BLIS from *Bacillus* isolates. Parameters included PEG, salt, and NaCl in the PEG/salt aqueous two-phase systems.

Parameters	*Bacillus velezensis* Y120-8			*Bacillus amyloliquefaciens* Y138-6			*Bacillus subtilis* Y167-2		
	Specific Activity (AU/mg)	Purification Factor	Yield (%)	Specific Activity (AU/mg)	Purification Factor	Yield (%)	Specific Activity (AU/mg)	Purification Factor	Yield (%)
Supernatant (Control)	189.4 ± 22.3	1	100	256.9 ± 31.4	1	100	243.1 ± 19.5	1	100
PEG MW (g/mol)									
1000	401.2 ± 46.8	2.1	93.5	732.3 ± 51.1	2.9	93.2	702.8 ± 54.5	2.9	89.1
3000	386.1 ± 26.1	2	81.3	694.8 ± 61.0	2.7	86.5	549.4 ± 51.2	2.3	78.6
4000	392.4 ± 31.8	2.1	81.7	655.4 ± 55.5	2.6	70.7	573.5 ± 35.1	2.4	73.1
10,000	336.0 ± 36.2	1.8	63.6	588.9 ± 47.8	2.3	46.5	445.3 ± 50.2	1.8	53.7
PEG concentration (%)									
10	101.6 ± 8.1	0.5	78.7	570.9 ± 43.5	2.2	60.7	495.1 ± 35.1	2	82.8
15	410.6 ± 17.4	2.2	88.3	817.2 ± 53.6	3.2	91.6	681.0 ± 41.4	2.8	82
20	337.0 ± 24.6	1.8	93.6	811.1 ± 44.1	3.2	93.4	613.1 ± 85.1	2.5	91.8
25	211.3 ± 15.3	1.1	82.5	700.6 ± 50.3	2.7	82.4	540.7 ± 33.8	2.2	84.6
Inorganic salt									
Na_3_C_6_H_5_O_7_	317.1 ± 31.3	1.7	88.6	659.4 ± 39.5	2.6	89.7	511.7 ± 50.2	2.1	80.1
(NH_4_)_2_SO_4_	383.6 ± 40.7	2	92.8	803.8 ± 45.1	3.1	92.6	680.9 ± 41.3	2.8	91.3
KH_2_PO_4_	165.3 ± 22.5	0.9	82.1	299.3 ± 34.9	1.2	84.7	383.6 ± 35.4	1.6	80.7
(NH_4_)_2_SO_4_ concentration (%)									
15	-	-	-	-	-	-	-	-	-
20	363.8 ± 42.1	1.9	93.8	810.5 ± 28.2	3	94.9	651.8 ± 33.3	2.6	93.7
25	313.3 ± 16.4	1.7	87.5	686.5 ± 21.5	1.1	76	611.6 ± 29.4	2.5	86.7
NaCl concentration (%)									
0%	393.9 ± 37.5	2.1	92.6	706.8 ± 40.9	2.9	95.5	622.9 ± 42.5	2.8	92.4
2%	588.1 ± 34.1	3.1	99.8	832.5 ± 27.5	3.2	100.1	756.7 ± 51.8	3.1	105.2
4%	121.8 ± 19.5	0.6	112	614.3 ± 31.6	2.4	116.3	556.0 ± 44.9	2.3	121.3
6%	84.9 ± 8.7	0.4	131.4	279.4 ± 26.9	1.1	122.7	267.2 ± 26.4	1.1	151.7

**Table 4:** Stability of BLIS partitioning at different pH values and temperatures.

Treatment		Remaining Activity (%)		
		*Bacillus velezensis* Y120-8	*Bacillus amyloliquefaciens* Y138-6	*Bacillus subtilis* Y167-2
Control (pH7, 37 °C, 15 min)		100 (432 AU/mg)	100 (825 AU/mg)	100 (730 AU/mg)
Temperature				
	50 °C, 15 min	100.80 ± 2.44	100.50 ± 3.64	99.69 ± 1.32
	60 °C, 15 min	92.59 ± 2.91	100.15 ± 1.80	96.44 ± 2.50
	70 °C, 15 min	81.48 ± 4.55	100.04 ± 2.31	87.44 ± 5.97
	80 °C, 15 min	68.98 ± 6.38	100.31 ± 1.79	55.24 ± 2.05
	90 °C, 15 min	46.53 ± 3.47	95.24 ± 2.38	40.40 ± 3.51
	100 °C, 15 min	26.62 ± 5.11	80.54 ± 4.51	13.34 ± 4.80
	121 °C, 15 min	0	0	0
pH				
	2	73.44 ± 2.55	81.25 ± 5.13	77.74 ± 4.15
	3	87.40 ± 4.03	99.44 ± 4.38	88.14 ± 3.32
	4	92.54 ± 3.78	100.29 ± 3.16	95.64 ± 1.55
	5	99.85 ± 1.16	100.45 ± 1.72	102.34 ± 2.95
	6	100.51 ± 1.62	102.08 ± 2.37	101.30 ± 1.44
	7	100.43 ± 1.35	100.71 ± 1.95	98.45 ± 2.04
	8	96.44 ± 2.21	97.37 ± 1.64	97.34 ± 4.33
	9	90.74 ± 3.44	92.81 ± 2.45	89.40 ± 2.21

## Data Availability

The data presented in this study are available in the article.
